# Supplementary material for: Brain-machine interface learning is facilitated by specific patterning of distributed cortical feedback
Source: Sci Adv. 2023 Sep 22;9(38):eadh1328. doi: 10.1126/sciadv.adh1328 (PMC10516504; doi:10.1126/sciadv.adh1328)
Supplement: Supplementary file 1 — Figs. S1 to S11 Table S1 [file sciadv.adh1328_sm.pdf]

Science Advances
AAAS

Supplementary Materials for

Brain-machine interface learning is facilitated by specific patterning of distributed cortical feedback

Aamir Abbasi *et al.*

Corresponding author: Valerie Ego-Stengel, valerie.ego-stengel@cnrs.fr

Sci. Adv. **9**, eadh1328 (2023)
DOI: 10.1126/sciadv.adh1328

This PDF file includes:

Figs. S1 to S11
Table S1

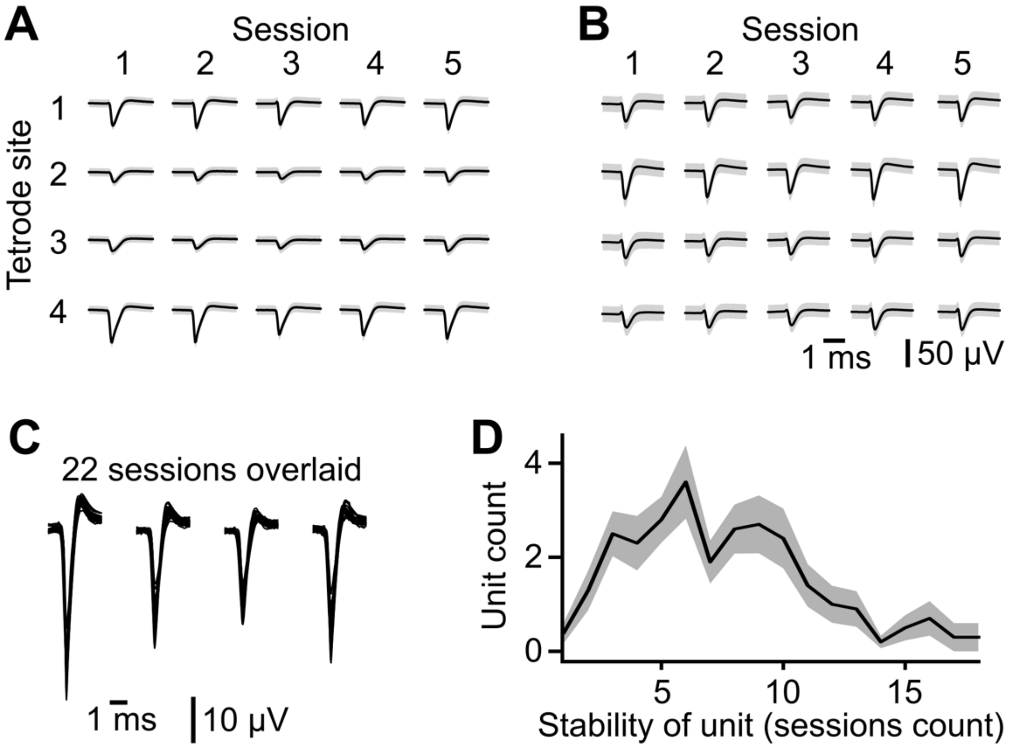

Fig. S1. Stability of the recorded neurons across sessions. (A) Stability of the spike shape of one Master neuron, shown for the 4 tetrode sites, across five training sessions, in the No feedback condition. **(B)** Same as a for a second Master neuron, this time in the Bar feedback condition. **(C)** Overlay of the spike shapes for one Master neuron across 22 successive sessions. **(D)** Average distribution across 10 mice of the count of sessions where the same Master unit could be identified. Shaded background: standard error of the mean of the histogram.

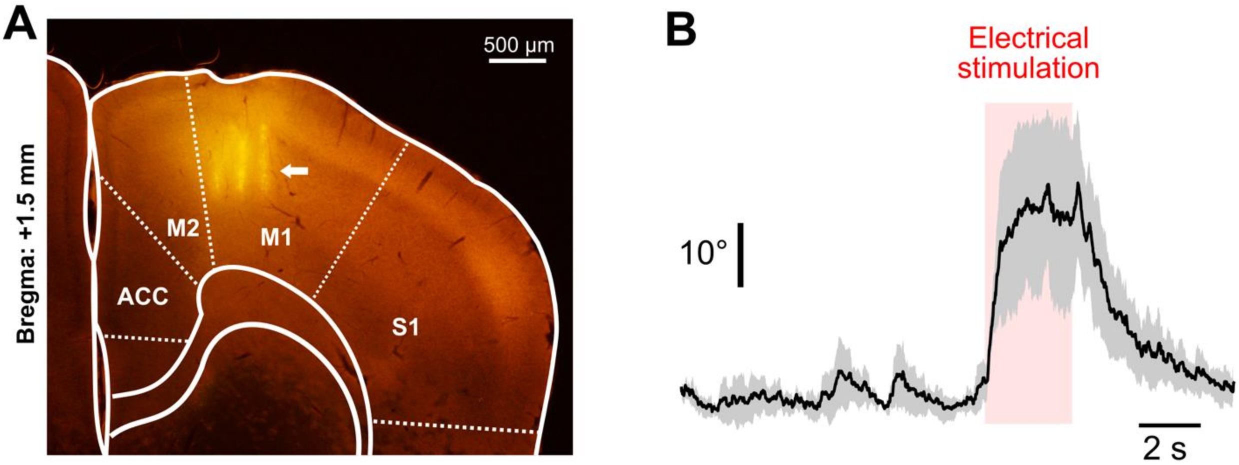

Fig. S2. Localization of the implanted silicon probes in whisker M1. **(A)** 50 µm coronal slice of a mouse brain, stained for Cytochrome oxidase. DiI coating of the shanks prior to insertion resulted in fluorescent lines indicative of the location of single shanks (yellow tracks) in M1 (white arrow). Dashed lines: area borders according to the Allen brain atlas. We identified the electrodes as being placed in the deeper layers of M1 based on: the location of the slice with respect to bregma; the lateral location of the electrode tracks with respect to the longitudinal fissure; and the depth of these tracks. **(B)** The amplitude of angular movements of a contralateral whisker evoked by intracortical microstimulation through the silicon probe (60Hz, 21 microA, average of 3 mice) confirms that the electrode was located in M1 (see Methods). Shaded background: standard error of the mean.

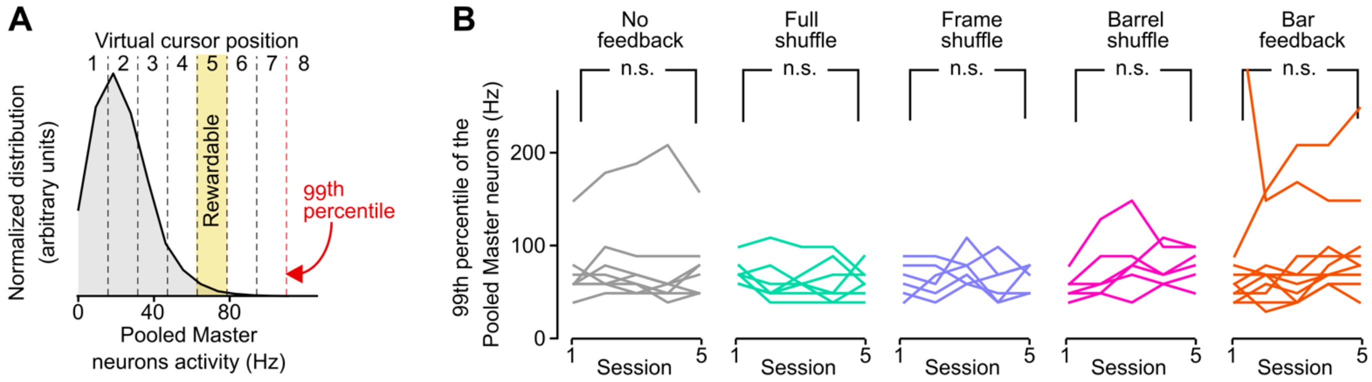

Fig. S3. Firing rate limits defining the eight possible positions of the virtual cursor. (A) Example distribution of the pooled firing rate of Master neurons during the 3 min baseline at the start of one session. The transition between virtual cursor positions 7 and 8 is set at the 99th percentile of the firing rate distribution. The range from 0 Hz to the 99th percentile is divided into equal firing rate intervals. Each firing rate interval corresponds to one virtual cursor position from 1 to 7 as indicated. **(B)** The 99th percentile of the pooled Master neurons firing rate did not evolve significantly over training sessions, regardless of the protocol. Each line: 1 mouse. (n.s.: Mann-Whitney $p > 0.05$).

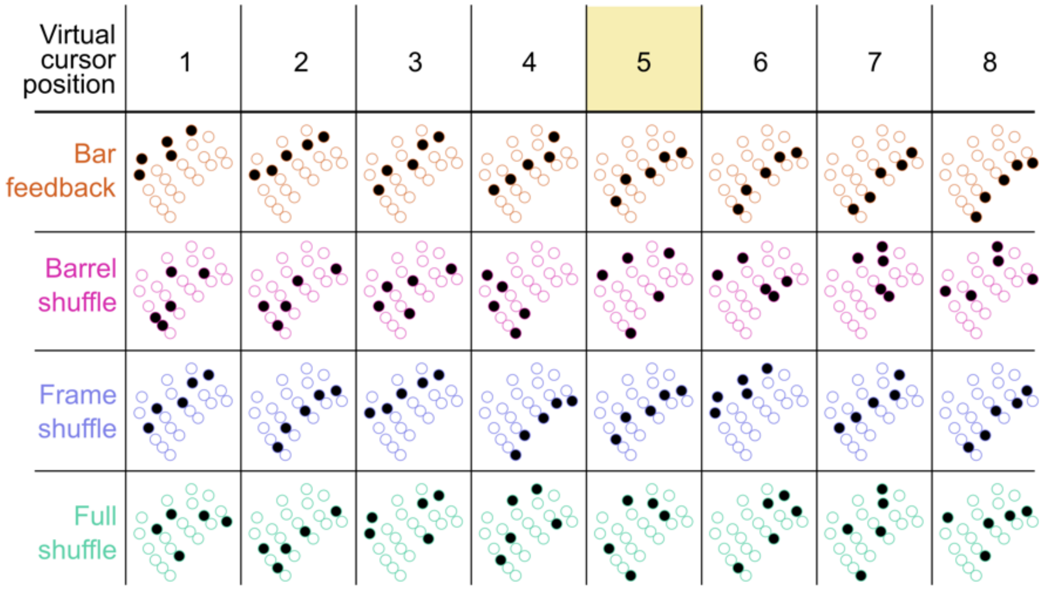

Fig. S4. Detail of the photostimulated barrels in the four feedback conditions, across the eight virtual cursor positions.

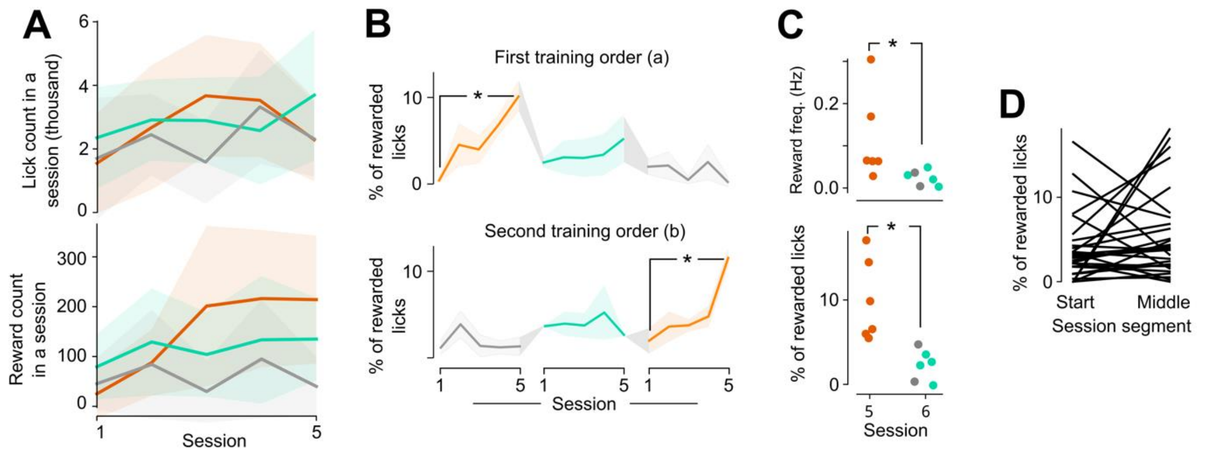

Fig. S5. Engagement and performance during training across feedback conditions. (A) Engagement of the mice during training sessions in the Bar feedback (orange), Full shuffle (green) and No feedback (gray) conditions. Left: total number of licks per session. Right: total number of rewards per session. Lines: average across mice. Shaded backgrounds: ± SEM. **(B)** Learning curves for two groups of mice (n = 3 in each group) trained with two different orders of presentation of three protocols: Bar feedback (orange), Full shuffle (green) and No feedback (gray). *: $p < 0.05$. Mann-Whitney non-parametric tests. Lines: average across mice. Shaded backgrounds: ± SEM. (**C**) Performance of the mice in the last session of training with the Bar feedback (orange), and performance on the following day when the mice transitioned either to No feedback (gray) or to Full shuffle feedback (green). The difference in performance is significant both in terms of reward frequency (Top) and percentage of rewarded licks (Bottom). *: Wilcoxon p = 0.031. Note that one mouse did not lick during the first session after the transition, so that we measured its performance in the second session after the transition. (**D**) Lack of visible intra-session learning. We measured the percentage of rewarded licks in two 300 s long windows, one at the start of the session, and a second 25 min later, measured during training sessions 2,3 and 4, in the Bar feedback condition. There was no significant difference (Wilcoxon p = 0.17).

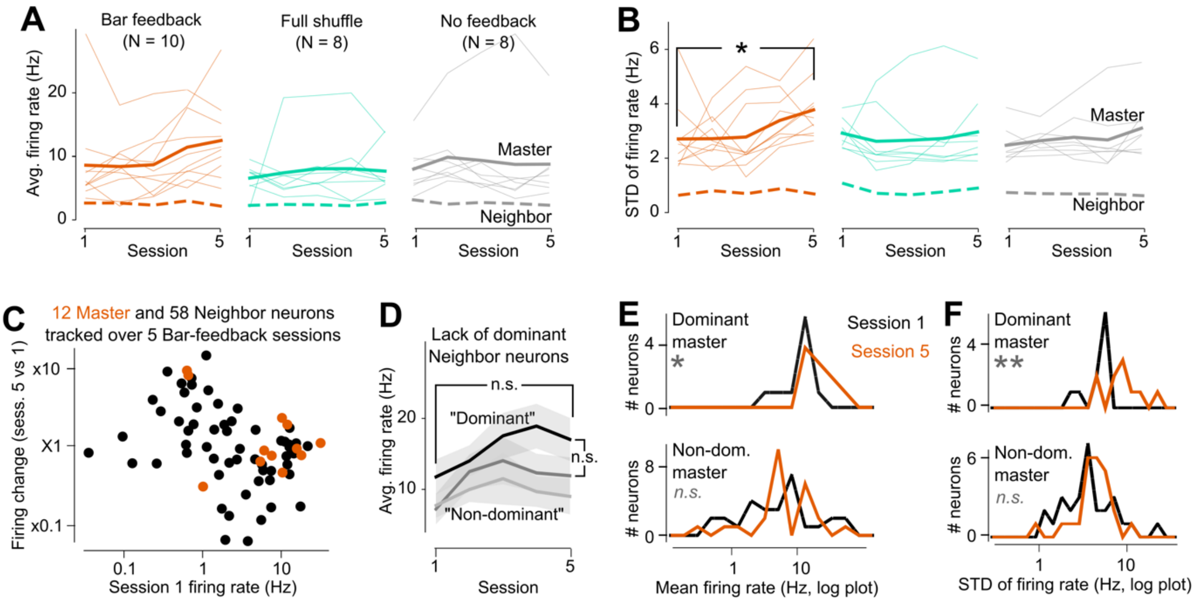

Fig. S6. Firing statistics of Master and Neighbor neurons, including comparisons between dominant and non-dominant neurons, during closed-loop learning. (A) Thin lines: Firing rate averaged over the individual Master neurons of one mouse as a function of training sessions. Thick lines: Average of individual neuron firing rates across all mice, for Master (continuous line) and Neighbor (dashed line) neurons. Bar feedback (orange), Ful shuffle (green) and No feedback (black) conditions. **(B)** Same as A, for the standard deviation of the firing rate (measured over 1-s windows). Mann-Whitney, *: $p < 0.05$. (**C**) Relationship between the firing rate of the Master neurons (orange) and Neighbor neurons (black) during the first training session (x axis) and the firing rate change between the first and fifth training session (y axis). Note that Master neurons have been arbitrarily selected on Session 1. Generally, neurons with low Session 1 firing rate tended to increase their firing rate, while neurons with high Session 1 firing rate did the contrary. Particularly, the two Master neurons which most increased their firing rate had the smallest firing rate initially. This analysis was restricted to neurons that we could record across the 5 training sessions in the Bar feedback condition (see Methods). (**D**) "Dominance" analysis of the Neighbor neurons recorded during the Bar feedback condition. We classified Neighbor neurons into one dominant neuron that showed the largest instantaneous firing rate around reward times and other non-dominant neurons (same analysis as for Master neurons). In contrast to Master neurons, we did not find that the mean firing rate of the dominant Neighbor neuron increased significantly over training sessions, ruling out an artefact of the dominance (Mann-Whitney $p > 0.05$). Note that the remaining trend for increasing firing rates could be explained by coupling between Master and Neighbor neurons. Light background: average over 10 mice. (**E**) Comparison of the mean firing rate of dominant versus non-dominant Master neurons across the 5 training sessions. Dominant Master neurons have a significantly larger firing rate on Session 5 compared to Session 1 (Mann-Whitney $p = 0.017$, $n = 10$ neurons). This was not the case for non-dominant neurons. (**F**) Same as E for the standard deviation, which increased significantly in dominant neurons with training (Mann-Whitney $p = 0.007$) but not in non-dominant neurons ($p = 0.17$). Interestingly, in Session 1 the standard deviation of the dominant neurons was not significantly different from the standard deviation of the non-dominant neurons, while in Session 5 it was highly significantly larger ($p = 5.4$ x 10-4).

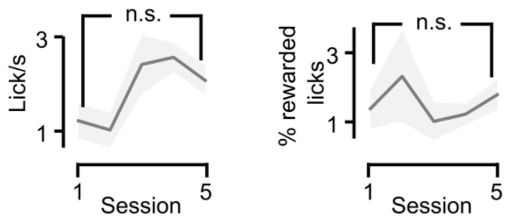

Fig. S7. Absence of learning during 5 sessions of bar playback training in five mice. Left: Mean (+/-SEM) frequency of licking per session. The increase was not significant. Right: Average (+/-SEM) proportion of rewarded licking over the 5 training sessions. The modulation is not significant. n.s: Mann-Whitney test $p > 0.05$.

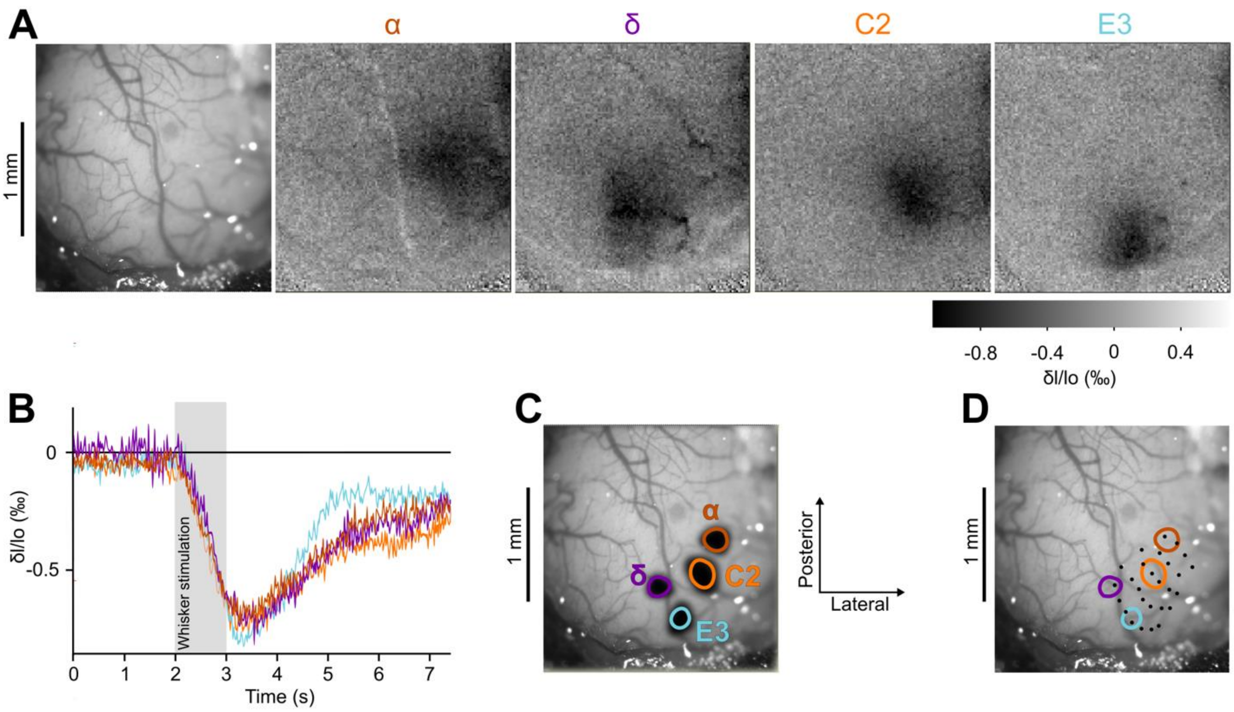

Fig. S8. Identification of the barrels position within the chronic optical window using intrinsic imaging shown for one mouse. (A) Image of the barrel cortex showing the blood vessels and intrinsic imaging during stimulations of the Alpha, Delta, C2 and E3 whiskers. **(B)** Time course of the whisker stimulation and of the intrinsic signal. Each whisker was stimulated for 1 second (grey area) with 100 Hz rostro-caudal deflections. **(C)** Thresholded contours of the intrinsic signal peaks used to define the location of the barrels. Contours of barrels correspond to 85% of the maximum relative absorption, after applying a 20th order gaussian filter. **(D)** Alignment of the barrel map from Knutsen and collaborators (see Methods) with the 4 barrels localized by intrinsic imaging.

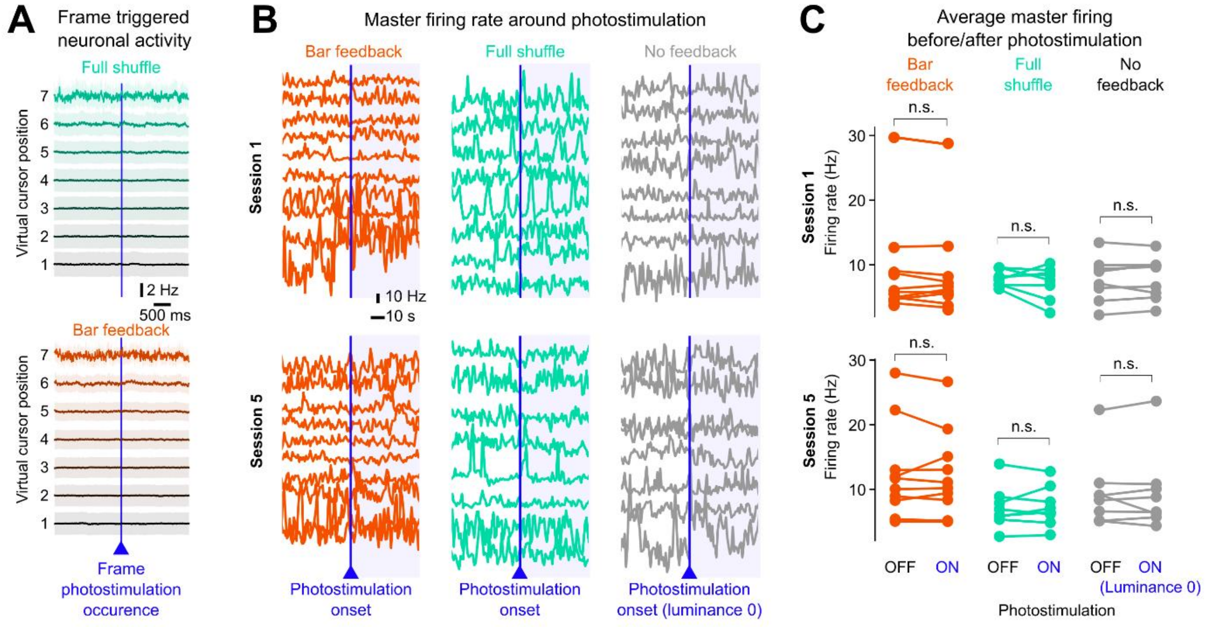

Fig. S9. Lack of direct impact of the photostimulation feedback in S1 on firing rates recorded in M1. (A) Mean +/- SEM of the summed activity of M1 neurons around the occurrences of photostimulation frames, during playback of Full shuffle and Bar feedback photostimulation sequences recorded in naïve animals (see Methods). Activity histograms are shown for the occurrence of virtual cursor positions 1 to 7, because position 8 does not occur frequently enough for this analysis. Top: Full shuffle (9 neurons, 3 mice). Bottom: Bar feedback (same neurons). Average firing rate of the neurons: 6.4 Hz. **(B)** Summed activity of the Master neurons (one line for each mouse) around the photostimulation onset for the first (top) and the fifth training session (bottom) for the Bar feedback, Full shuffle and No feedback conditions. Vertical blue lines: onset of the photostimulation, which then goes on uninterrupted for the whole session (light blue background). The Frame shuffle and Barrel shuffle feedback conditions gave similar results. **(C)** Average Master neuron firing rate across a subset of mice, in the 180 s before/after the onset of the photostimulations, for the first session (top) and the last session (bottom). In all three conditions tested, the Wilcoxon paired test p-value was not significant ($p > 0.05$). For all panels: Orange: Bar feedback. Green: Full shuffle. Black: No feedback.

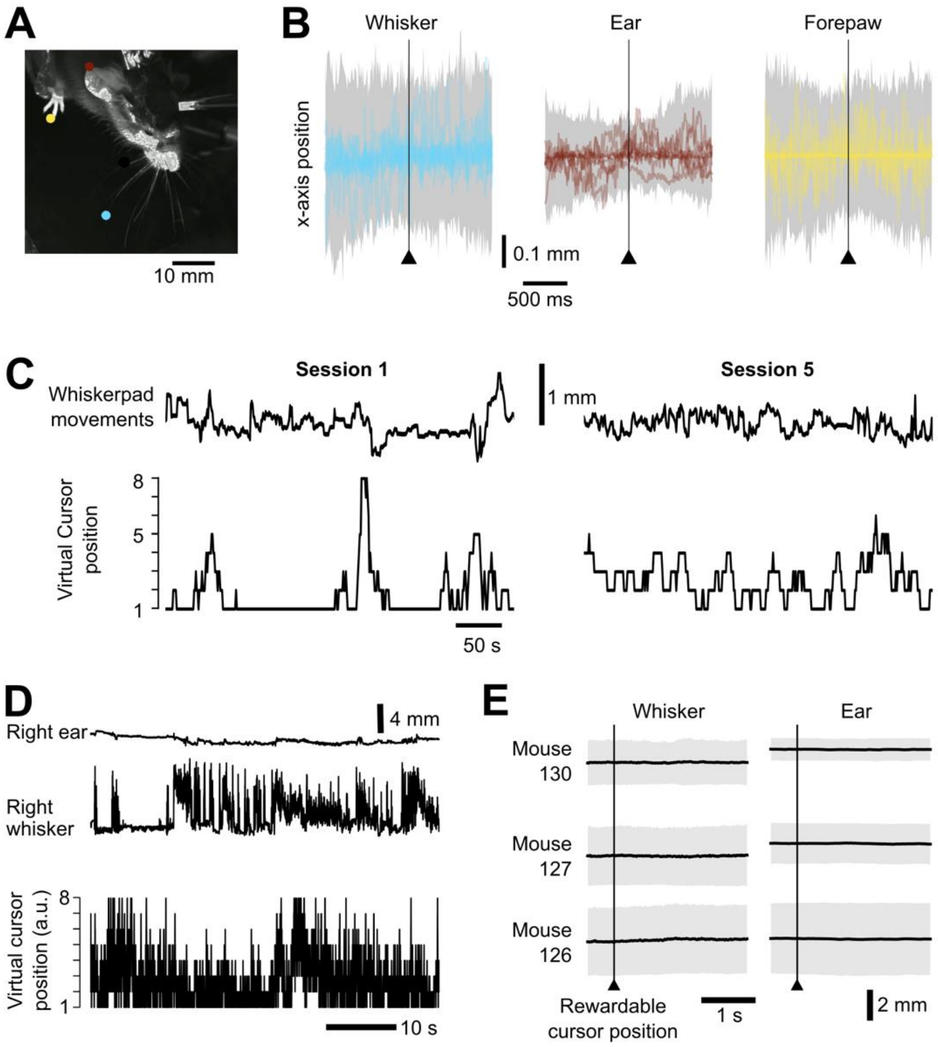

Fig. S10. Relationship of brain-machine interface M1 readout with movements of the mouse. (A) Tracking of several body parts positions using DeepLabCut, including a whisker (blue), the right forepaw (yellow) and the right ear (red). **(B)** Individual traces correspond to the average spike-triggered movement of the body parts selected in the picture in **A**, for each individual Master neurons of 3 mice, during a session of spontaneous behavior that followed immediately a series of brain-machine interface training sessions. Gray background: bootstrap-based 1% significance threshold for the spike-triggered analysis of the movements. Left: Whisker movements. Middle: Ear movements. Right: Forepaw movement. (**C**) Examples of whisker movements during the first versus fifth training session in one example mouse in the Barrel shuffle condition. Top: position of the base of one of the tracked whiskers measured with DeepLabCut. Bottom: virtual cursor position computed from ongoing M1 neuronal activity. (**D**) Top: Example of movements of one right whisker and the right ear during a session of Bar playback, taking place after 5 sessions of Bar feedback sessions. Bottom: position of the optogenetic cursor. The ear and whisker movements are shown on the axis where the movement is largest. Note the lack of relationship between the whisker movements and the virtual cursor position. (**E**) Average whisker and ear movements, aligned on the entrance of the virtual cursor on the rewardable position. Light background: standard deviation.

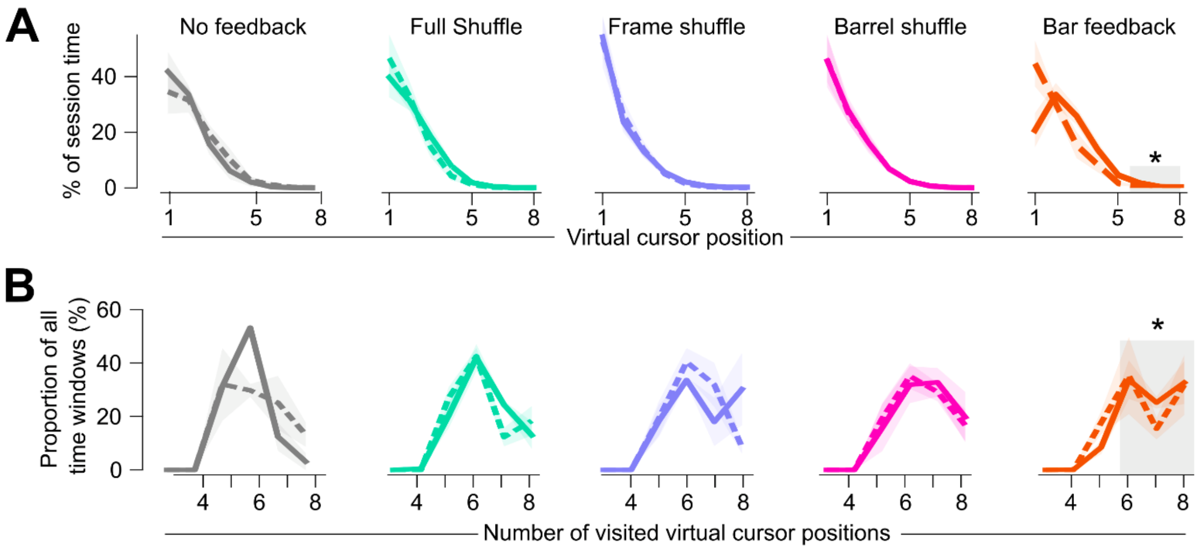

Fig. S11. Changes in the exploration of the 8 positions of the virtual cursor, at the beginning and at the end of training. (A) Percentage of time spent by the cursor in the different positions, in Session 1 (Dashed line) versus Session 5 (Continuous line), across the 5 feedback conditions tested. Light background: standard error of the mean across mice. There was a significant increase in the proportion of time spent in positions 6, 7 and 8 in the Bar feedback condition (Mann-Whitney $p = 0.037$). **(B)** Percentage of time windows (+/- 2 s centered on reward times) as a function of the number of positions visited by the virtual cursor in those time windows, on Session 1 (Dashed line) versus Session 5 (Continuous line). Only sessions with more than 10 rewards were considered. Light background: standard error of the mean across mice. After 5 training sessions, the virtual cursor visited at least 6 of the 8 cursor positions in 92% of these time windows, a significant increase from the 82% observed in Session 1 (Mann-Whitney $p = 0.043$). In other words, this means that during the 4 seconds of a given reward-centered window, the cursor spent some amount of time in each of 6 cursor positions, for example positions 1 to 6. This was true for 92% of the time windows, while in the remaining 8%, the cursor may have stayed static during the full 4 seconds or explored fewer different positions.

Mouse	Session																
	1	2	3	4	5	6	7	8	9	10	11	12	13	14	15	16	17
1	R	R	R	R	R	F	F	F	F	F							
2	R	R	R	R	R	F	F	F	F	F							
3	R	R	R	R	R	F	F	F	F	F							
4	F	F	F	F	F	R	R	R	R	R							
5	F	F	F	F	F	R	R	R	R	R							
6	F	F	F	F	F	R	R	R	R	R							
7	B	B	B	B	B	N	N	N	N	N							
8	B	B	B	B	B												
9	B	B	B	B	B	U	U	U	U	U							
10	B	B	B	B	B	U	U	U	U	U	N	N	N	N	N		
11	B	B	B	B	B	U	U	U	U	U	N	N	N	N	N		
12	B	B	B	B	B	U	U	U	U	U	N	N	N	N	N		
13	U	U	U	U	U	B	B	B	B	B	N	N	N	N	N		
14	N	N	N	N	N	U	U	U	U	U	B	B	B	B	B	B	P
15	N	N	N	N	N	U	U	U	U	U	B	B	B	B	B	B	P
16	N	N	N	N	N	U	U	U	U	U	B	B	B	B	B	B	P

Table S1. Identity of the feedback patterns presented to the mice across successive training sessions. Mice are ordered by similarity of protocols. Bar Feedback: **B**, Full Shuffle: **U**, No Feedback: **N**, Barrel shuffle: **R**, Frame shuffle: **F**, Bar Playback: **P**
